# Catalytic Valorization of Palm-Based Fatty Acids into High-Performance Estolide Ester Lubricants

**DOI:** 10.1021/acsomega.6c05480

**Published:** 2026-09-24

**Authors:** Paphada Watcharapo, Pawnprapa Pitakjakpipop, Wipark Anutrasakda, Harit Pitakjakpipop

**Affiliations:** † National Energy Technology Center, 61191 National Science and Technology Development Agency (NSTDA), 114 Thailand Science Park, Phahonyothin Road, Klong 1, Klong Luang, Pathum Thani 12120, Thailand; ‡ Department of Chemistry, Faculty of Science, Chulalongkorn University, Payathai Road, Patumwan, Bangkok 10330, Thailand; § National Nanotechnology Center, National Science and Technology Development Agency (NSTDA), 111 Thailand Science Park, Phahonyothin Road, Klong 1, Klong Luang, Pathum Thani 12120, Thailand

## Abstract

Lubricants derived from petroleum, a nonrenewable resource, raise increasing concerns regarding environmental impact and long-term sustainability, driving demand for renewable alternatives. Vegetable oils are promising candidates; however, their direct use is limited by poor oxidative stability and inadequate low-temperature performance. Herein, palm-derived oleic acid and lauric acid were catalytically valorized into estolide esters via a one-pot process using Amberlyst-15 as a heterogeneous sulfonic acid catalyst. Estolide formation followed by esterification with 2-ethylhexanol proceeded efficiently under vacuum, with the catalyst retaining its ion-exchange capacity and chemical composition after consecutive steps. The effects of reaction temperature, mole ratio, and catalyst amount on estolide synthesis were examined. The resulting oleic–lauric estolide esters (OL-EST-EH) exhibited favorable lubricant-relevant properties, including a high viscosity index (158–169), excellent cloud point below −25 °C, pour point below −27 °C, and oxidative stability of up to 20.3 h under accelerated testing at 140 °C. These results highlight OL-EST-EH as a promising biobased base oil candidate for formulating high-performance, environmentally sustainable lubricants.

## Graphical abstract

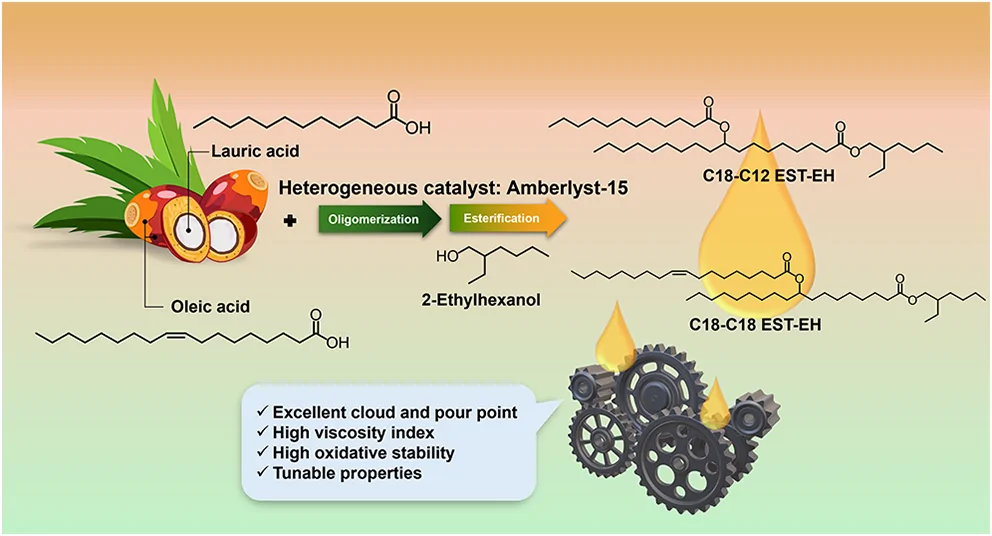

## 1 Introduction

Lubricants are widely used in various applications, such as automotive, machinery, and processing, to reduce friction and wear, assist cleaning, provide corrosion protection, and improve heat transfer. Conventionally, they are currently produced through petroleum refining, a process that relies on nonrenewable energy sources. Petroleum-based lubricants can negatively impact human health and the environment due to their low biodegradability, high toxicity and contribution to global warming.^1^ As lubricant consumption increases, awareness of environmental preservation is driving a global shift toward sustainable strategies to transition from fossil-based oils to environmentally friendly alternatives.

Lubricants typically consist of base oils formulated with additives to enhance their performance. Base oils are generally classified into mineral, synthetic, and other categories, including vegetable oils. Vegetable oils are promising candidates for biolubricants and represent attractive substitutes for conventional base oils because they are biodegradable, derived from renewable resources, and exhibit favorable lubricating properties, such as high viscosity index, high flash point, and low friction coefficient.^2^ In addition, the triglycerides in vegetable oil exhibit higher polarity than mineral-based lubricants, thereby enabling greater attraction to metal surfaces.^3^ However, using vegetable oil directly as a base oil is limited by the presence of a hydrogen atom on the β-carbon of an ester molecule and by unsaturation in the fatty acid chain, which leads to poor oxidation and thermal stability.^4,5^ Key lubricant performance requirements include high thermal and oxidative stability, a high viscosity index, a high flash point, and low pour and cloud points. Previous studies have shown that the limitations of vegetable oils can be improved by blending with additives and/or chemical modification.^6,7^ Additives are commonly used to enhance lubricant performance by improving resistance to oxidation and hydrolysis, controlling viscosity, inhibiting corrosion and rust formation, and enhancing low-temperature performance.

The common methodologies used to synthesize biolubricants from vegetable oil include transesterification/esterification, epoxidation, hydrogenation, and estolide formation, all of which aim to achieve lubricating properties comparable to or superior to those of mineral-based oils.^8–11^ Vegetable oils are primarily composed of triglycerides, which are glycerol esters of three linear fatty acids, typically ranging from C12 to C20.^7^ The chain length and degree of unsaturation of these fatty acids play critical roles in determining the physicochemical properties of the resulting biolubricants. Common feedstocks for biolubricant synthesis include palm, canola, soybean, sunflower, coconut, jatropha, castor, and lesquerella oils.

One widely applied route involves the esterification of fatty acids (RCOOH) derived from vegetable or seed oils with polyhydric alcohols (ROH) to produce polyol esters.^12–15^ Another approach used for more than a decade, but still challenging, is estolide formation. Estolides are generated when the carboxylic acid group of one fatty acid reacts with the carbon–carbon double bond of another unsaturated fatty acid, forming an ester linkage. The resulting structure is characterized by the estolide number (EN), defined as *n* + 1, where *n* represents the average number of fatty acid units attached to a base fatty acid; when *n* = 0, a dimer is formed.^16,17^ The oligomeric ester structure is more resistant to hydrolysis than triglycerides and improves the physical properties of the lubricant, including increased oxidative stability, viscosity index, and a lower pour point.^18,19^ The structure and properties of estolides are strongly influenced by the chain length and degree of unsaturation of the constituent fatty acids. Cermak and Isbell reported the synthesis of estolides using oleic acid as a base unit and various saturated fatty acids as capping agents in the presence of perchloric acid as a catalyst.^20^ The EN was impacted by the chain length of the saturated fatty acid used as a capping agent. It was observed that short-chain saturated fatty acids produced higher degrees of estolide formation compared to long-chain fatty acids due to steric hindrance or p*K*_a_ effects. Furthermore, the chain length of saturated fatty acids influences their physical properties, including pour point, cloud point, and viscosity. A series of saturated-capped estolides was distilled to remove excess free fatty acids and byproducts and then esterified with 2-ethylhexanol to form estolide 2-ethylhexyl esters. The physical properties of these estolides and their esters were reported.^21^ The short-chain saturated-capped estolide 2-ethylhexyl esters exhibited better low-temperature properties than those with longer chain lengths and commercial lubricants. Furthermore, viscosity was significantly influenced by the degree of oligomerization. This study found that free acid estolides exhibited higher viscosities than their corresponding esters due to hydrogen bonding of the carboxyl group. Meanwhile, estolide 2-ethylhexyl esters exhibited a higher viscosity index than the free estolide, indicating that the fluid exhibits less viscosity change over a wide temperature range, which is critical for high-performance lubrication applications.

Estolide formation is typically promoted by catalysts to enhance reaction rates and control product selectivity, employing either homogeneous or heterogeneous systems. Previous studies have investigated various homogeneous acid catalysts for estolide synthesis, including perchloric acid (HClO_4_), sulfuric acid (H_2_SO_4_), methanesulfonic acid (CH_3_SO_3_H), and nitric acid (HNO_3_).^22^ Among these catalysts, HClO_4_ exhibited the highest depletion of carboxylic group content, indicating its superior catalytic performance in promoting estolide formation. Perchloric acid and sulfuric acid have also been reported as widely employed homogeneous catalysts for this reaction.^23–25^ Homogeneous catalysts generally offer high reactivity and selectivity because the catalyst and reactants are present in the same phase, often leading to high product yields.^26,27^ It also has an industry advantage, allowing it to operate under milder conditions (lower temperatures and pressures) and reduce energy costs.^28^ However, the primary challenge with this catalyst is that it is corrosive to the reactor, nonreusable, and difficult to separate from the products, which requires an additional purification step. In contrast, heterogeneous catalysts, including the commercially available immobilized lipase Novozym 435 ^29^ and modified clay catalysts,^30^ have been explored as alternatives for estolide synthesis and offer advantages in catalyst recovery and reusability due to their distinct phase from the reaction mixture. Nevertheless, their performance can be limited by mass transfer resistance, as reactions occur at surface active sites, potentially resulting in lower conversions compared to homogeneous systems.^31,32^ For this reason, solid catalysts require more severe reaction conditions, including a higher alcohol-to-oil molar ratio, greater catalyst loading amount, and elevated temperatures, to achieve the same level of conversion.^31^ Despite these limitations, heterogeneous catalysis remains attractive for sustainable and industrial processes due to its operational and environmental benefits.

Among renewable feedstocks, palm oil represents an economically significant crop and a major source of vegetable oil production in Thailand. Commercially available purified oleic acid and lauric acid, which are commonly derived from palm oil and palm kernel oil, respectively, were selected as model feedstocks in this study. Oleic acid, the unsaturated fatty acid in palm oil, is widely used as the base unit for estolide synthesis,^11,33,34^ whereas lauric acid, the predominant saturated fatty acid in palm kernel oil, serves as an effective capping unit. The use of purified fatty acids provides a well-defined reaction system, minimizing feedstock compositional variability and enabling systematic evaluation of catalytic performance and product structure–property relationships. In this study, these two fatty acids were utilized as feedstocks for a one-pot estolide formation and subsequent esterification with 2-ethylhexanol. Although various catalytic systems have been explored for estolide ester synthesis, homogeneous acid catalysts remain the predominant catalytic systems reported for estolide synthesis,^35,36^ whereas other synthetic routes require multiple chemical modification steps of fatty acid feedstocks to produce estolide esters.^24,37^ These approaches may increase process complexity and challenges for catalyst separation, process integration, and practical scalability. Consequently, the development of an efficient heterogeneous catalyst capable of integrating estolide formation and esterification into a simplified process remains an important research challenge. Although Amberlyst-15 has been widely employed as a heterogeneous acid catalyst for esterification and biodiesel production,^38,39^ its catalytic properties make it a promising candidate for the proposed one-pot synthesis strategy. Therefore, it was employed in this study to facilitate the one-pot sequential transformation of fatty acid feedstocks into estolide ester-based lubricant base oils. Compared with previously reported homogeneous acid-catalyzed systems or multistep synthetic routes requiring chemical premodification of fatty acid feedstocks, the proposed approach simplifies the synthetic pathway while facilitating catalyst separation and offering the potential for catalyst reuse. The effects of reaction temperature, oleic-to-lauric acid molar ratio, and catalyst loading were systematically investigated to elucidate structure–property relationships. The resulting products were characterized, and their lubricant-relevant properties were evaluated in accordance with ASTM International (formerly American Society for Testing and Materials) standards, an international organization that develops consensus-based testing methods, serving as benchmarks for biolubricant development. This work demonstrates a practical and sustainable catalytic strategy for upgrading palm-derived fatty acids into high-performance lubricant base oils for industrial applications.

## 2 Experimental Section

### 2.1 Materials

Lauric acid (C12:0, 99%) was purchased from Chemipan Corporation Co., Ltd. (Bangkok, Thailand). Oleic acid (C18:1, Extra Pure), *n*-hexane (AR grade), sodium chloride (AR grade), potassium hydroxide pellets (AR grade), toluene (AR grade), and ethyl acetate (AR grade) were purchased from QReC, New Zealand. Amberlyst-15 (Dry) was purchased from Sigma-Aldrich. 2-Ethyl-1-hexanol (>99.5%) and *N*,*O*-bis­(trimethylsilyl)­trifluoroacetamide (BSTFA, >95.0%) were purchased from Tokyo Chemical Industry Co., Ltd. Isopropyl alcohol (≥99.8%) was purchased from Fisher Chemical.

### 2.2 Catalyst Characterization

The surface morphologies and elemental composition of Amberlyst-15 were examined using a field-emission scanning electron microscopy (FE-SEM, JEOL JSM-7800FPRIME) equipped with energy-dispersive X-ray spectroscopy (EDS, Oxford Instruments). Before imaging, the samples were sputter-coated with a thin layer (5–10 nm) of gold to improve conductivity. The used catalyst was washed four times with *n*-hexane and dried at 80 °C for 2 h prior to analysis. The titration method was used to determine the ion-exchange capacity (IEC) of Amberlyst-15. 0.1 g of dried resin was accurately weighed and added to 25 mL of 2 M NaCl solution. The mixture was stirred at 350 rpm for 30 min to allow the exchange of Na^+^ ions in the solution with H^+^ from the sulfonic groups on the resin surface. Then, the amount of exchanged H^+^ was titrated with 0.1 M potassium hydroxide (KOH) solution using an automatic titrator (T50, METTLER TOLEDO), and the experiment was conducted in duplicate. The volume of KOH solution was recorded at the end point (pH = 8.3). The IEC, expressed in milliequivalents per gram (meq/g), was calculated using the formula:

$$\mathrm{IEC}=\frac{cv}{w}$$

where *c* is the standardized concentration (M), *v* is the volume at the end point of the KOH solution (mL), and *w* is the weight of dried resin.

### 2.3 Synthesis of OL-EST and OL-EST-EH

The one-pot synthesis of estolide esters was carried out through consecutive oligomerization and esterification reactions to produce estolides and estolide esters, respectively. The experimental setup consisted of a 500 mL three-neck round-bottom flask equipped with a condenser and a magnetic stirrer. A thermocouple was used to monitor the temperature, while a hot plate with a silicone oil bath provided heating and temperature control. A vacuum pump with an adjustable valve was employed to regulate the vacuum level throughout the experiment. For the oligomerization step, oleic acid (O) and lauric acid (L) were homogeneously mixed by heating at 60–80 °C under vacuum with stirring for 30 min. Once the mixture reached the target temperature, Amberlyst-15 was added to initiate the reaction, and the vacuum was maintained at 80 mbar while stirring continued for 24 to 48 h. This study specifically examined the effect of synthesis conditions, including reaction temperatures (60, 80, 90, and 120 °C), oleic-to-lauric acid mole ratios (0.25:1, 0.5:1, 1:1, 2:1, and 1:0), and catalyst loadings (10, 20, and 30 wt %). The resulting oleic-lauric estolides (OL-EST) were characterized using FT-IR, GC-FID, GC-MS, and ^1^H NMR. Following oligomerization, 2-ethylhexanol (2-EH) was added for esterification of OL-EST at 80 °C under 20 mbar vacuum for 24 h to produce oleic-lauric estolide esters (OL-EST-EH). The catalyst was removed by vacuum filtration through a Büchner funnel using Whatman grade 5 filter paper (pore size 2.5 μm). The liquid products were then distilled under vacuum at 80–100 °C to remove excess 2-EH, producing crude oleic-lauric estolide esters (crude OL-EST-EH). Further purification was performed by vacuum distillation at 150–200 °C to separate byproducts and monoesters, resulting in the final distilled OL-EST-EH product (dis.OL-EST-EH). The crude product recovery was 85–87%. The recovery below 100% is mainly attributed to the removal of water generated during the esterification reaction under vacuum, with a minor loss of excess 2-EH occurring due to evaporation under the reaction conditions. GC-FID analysis showed that the crude product consisted of 27–29% estolide esters, 69–71% fatty acid esters, and < 2% residual free fatty acid. After distillation to remove the light fraction, the distilled estolide ester fraction (dis.OL-EST-EH) was obtained with a purity of 85–88%, as determined by GC-FID.

### 2.4 Equipment and Procedures

#### 2.4.1 Proton Nuclear Magnetic Resonance (^1^H NMR)

^1^H NMR spectroscopy was employed to characterize the proton environments in OL-EST and its byproduct using a JEOL JNM-ECZ500R/S1 NMR spectrometer operating at 500 MHz. Deuterated chloroform (CDCl_3_) was used as the solvent for all samples. The estolide number (EN) was determined from the integral ratio of α-ester to α-acid protons.^20,40,41^

#### 2.4.2 Fourier Transform Infrared Spectroscopy (FT-IR)

The chemical functional groups of feedstock and product were characterized by Nicolet iS50 FTIR spectrometer (Thermo Fisher Scientific). The samples were recorded in attenuated-total-reflection (ATR) mode, with a resolution of 4 cm^–1^ and 64 scans covering wavenumbers between 500 and 4000 cm^–1^.

#### 2.4.3 Gas Chromatography Flame Ionization Detection (GC-FID)

The composition of free fatty acids and products was analyzed using an Agilent 7890B gas chromatograph (Agilent Technologies, Inc.), equipped with a flame-ionization detector (FID). Analysis was performed on a DB-5HT column (15 m × 0.32 mm, 0.10 μm). The GC-FID operating conditions were as follows: column flow, 1 mL/min; split ratio, 100:1; oven program, 90–170 °C at 5 °C/min, then 170–320 °C at 10 °C/min, with a final hold of 10 min at 320 °C; injector and detector temperature, 300 and 380 °C, respectively. A 0.04 g sample was silylated with 150 μL of *N*,*O*-bis­(trimethylsilyl)­trifluoroacetamide (BSTFA) as a silylation agent. After silylation, 2 mL of ethyl acetate was added as the solvent. Pure lauric acid and oleic acid were used as standards for quantification. The composition and conversion were calculated using the following equations:

$$\%\;\mathrm{composition of FFA}=\frac{A_{\mathrm{FFA}}}{A_{\mathrm{STD}}}\times 100$$

%composition of product=100−%composition of FFA

$$\%\;\mathrm{product selectivity}=\frac{\mathrm{area of product}}{\mathrm{total area of product}}\times 100$$

$$\mathrm{crude product recovery}\;\left(\%\right)=\frac{m_{\mathrm{crude product}}}{m_{\mathrm{oleic acid}}+m_{\mathrm{lauric acid}}+m_{2\mathrm{‐EH}}}\times 100$$

$$\%\;\mathrm{conversion}=\frac{F_{0}-F_{t}}{F_{t}}\times 100$$

where *A*_FFA_ is the peak area of free fatty acids in the product, *A*_STD_ is the peak area of free fatty acids in the feedstock, and *F*_0_ and *F*_t_ represent the composition (%) of free fatty acids before and after synthesis, respectively.

#### 2.4.4 Total Acid Number (TAN) or Acid Value

The acid value was determined using an automatic titrator (Mettler-Toledo T50) according to ASTM D664. An accurately weighed sample was dissolved in 60 mL of a solvent mixture consisting of toluene, isopropyl alcohol, and deionized water at a volume ratio 50.0:49.5:0.5, and then potentiometrically titrated with a standardized 0.1 M KOH in isopropyl alcohol. The acid value was calculated using the following equation:

$$\mathrm{acid value}\;(\mathrm{mg KOH}/g)=\frac{56.1C_{\mathrm{KOH}}V_{\mathrm{KOH}}}{m}$$

where 56.1 is the molecular weight of KOH (g/mol), *C*_KOH_ is the concentration of KOH titrant (g/mol), *V*_KOH_ is the volume of KOH solution used (mL), and *m* is the weight of the sample (g).

#### 2.4.5 Cloud Point and Pour Points (CP and PP)

The cloud point and pour point were measured by a mini pour and cloud point tester (mini-MPC-102S analyzer, Tanaka Scientific Limited). The cloud point (CP) was determined at 1 °C intervals using the small-test-jar method (ASTM D7683), comparable to ASTM D2500. The pour point (PP) was determined at 1 °C intervals by the air pressure method (ASTM D6749), comparable to ASTM D97 and ASTM D5949. Both measurements can be performed simultaneously in a single run using 4.5 mL of sample with a reflex seal on the bottom of the cloud point test cell.

#### 2.4.6 Differential Scanning Calorimetry (DSC)

The crystallization and melting behavior of the synthesized oils was analyzed using DSC with a Shimadzu DSC-60 Plus instrument. Nitrogen was used as the purge gas at a flow rate of 20 mL/min. Approximately 5 mg of each sample was weighed into an aluminum pan. The thermal program consisted of holding the sample at 50 °C for 10 min, cooling to −85 °C at 5 °C/min to obtain the cooling profile, and then heating from −85 to 50 °C at the same rate to obtain the melting profile.

#### 2.4.7 Viscosity and Viscosity Index (VI)

Kinematic viscosity was measured at 40 and 100 °C using an automated kinematic viscosity measurement viscometer (miniAV, Cannon Instrument Company) in accordance with ASTM D445. The viscosity index was calculated following ASTM D2270.

#### 2.4.8 Oxidative Stability

The oxidative stability of the products was evaluated under accelerated conditions using a Rancimat instrument (Metrohm 743, Herisau, Switzerland). The analysis was performed using 3.0 ± 0.1 g of liquid sample at 140 °C under an air flow of 20 L/h. The result was expressed as induction time (h), defined as the period during which the sample resisted oxidative degradation, indicated by a rapid increase in the conductivity of deionized water.

## 3 Results and Discussion

### 3.1 Physicochemical Properties of the Catalyst

To evaluate the stability of Amberlyst-15 catalyst after OL-EST and OL-EST-EH synthesis, SEM was conducted on both the fresh and used catalysts, as shown in Figure S1. The fresh Amberlyst-15 particles exhibited diameters of approximately 600–850 μm, with minor surface cracks. In comparison, the used catalyst showed no significant changes in particle size or surface morphology. Moreover, no swelling was observed after *n*-hexane washing, confirming the structural stability and potential reusability of Amberlyst-15. Moreover, the elemental composition of fresh and used Amberlyst-15 catalysts was analyzed using SEM-EDS. The catalysts were primarily composed of carbon, oxygen, and sulfur, corresponding to the sulfonic acid groups that function as active sites (Table S1). A slight decrease in sulfur content was observed after both the first and second consecutive uses, compared with the fresh catalyst. Notably, this sulfur content was not affected by *n*-hexane washing. These results indicate that Amberlyst-15 maintained its chemical composition and catalytic activity after two consecutive reaction steps.

The ion-exchange capacity (IEC) of Amberlyst-15 was determined by acid–base titration to evaluate changes in the catalyst acidity after its use as a catalyst in one-pot synthesis of biolubricant base oils via consecutive estolide formation and esterification (Table 1). Amberlyst-15 is a solid acid catalyst in which the sulfonic acid groups (−SO_3_H) serve as the active sites for catalysis. In this study, a sodium chloride solution was used to exchange H^+^ ions from sulfonic groups with Na^+^ ions. The used catalyst was washed with a *n*-hexane solution and dried prior to IEC analysis. The IEC indicates catalytic activity in the synthesis reaction. Typically, Amberlyst-15 exhibits a high IEC, reported to be approximately 4.7–5.1 meq/g on a dry weight basis^42^,which is consistent with the result obtained in this work. Fresh Amberlyst-15, both with and without *n*-hexane washing, showed similar IEC values, with the washed fresh catalyst serving as a control to confirm that *n*-hexane washing did not significantly affect IEC. After the reaction, the IEC decreased due to ion exchange with free fatty acids, and it was only slightly lower after the second step than after the first. Catalyst reusability was further evaluated over three consecutive reaction cycles using the same catalyst (one initial use followed by two reuse cycles), where each reaction cycle consisted of two consecutive reaction steps (estolide formation followed by esterification). As summarized in Table 1, the IEC gradually decreased with repeated use of Amberlyst-15. Nevertheless, no significant changes in product composition were observed over the three reaction cycles (Table S2), indicating that the remaining exchangeable acid sites were sufficient to maintain the catalytic performance under the investigated conditions. Previous studies have reported that water produced during the esterification reaction can adversely affect the acid sites of Amberlyst-15.^43^ In the present study, however, the esterification reaction was performed under vacuum, allowing continuous removal of the generated water, thereby shifting the reaction equilibrium toward ester formation while helping to preserve the catalyst acidity. Consequently, Amberlyst-15 maintained satisfactory catalytic performance throughout six consecutive reaction steps without catalyst regeneration, requiring only solvent washing between reaction cycles. Although additional recycling studies over more reuse cycles would provide further insight into the catalyst’s long-term stability, the present results demonstrate satisfactory short-term operational stability and support the applicability of Amberlyst-15 in the proposed one-pot catalytic process.

**Table 1:** IEC of Fresh and Reused Amberlyst-15 Catalysts Determined by Titration

sample	IEC (meq/g)
fresh Amberlyst-15	4.73 ± 0.05
fresh Amberlyst-15 washed with *n*-hexane	4.78 ± 0.02
used Amberlyst-15 after OL-EST synthesis	4.45 ± 0.03
used Amberlyst-15 after OL-EST and OL-EST-EH synthesis	4.35 ± 0.02
used Amberlyst-15 after the 2nd cycle	4.07 ± 0.18
used Amberlyst-15 after the 3rd cycle	3.80 ± 0.07

### 3.2 Composition of Model Fatty Acid Feedstocks

For this study, OL-EST-EH was synthesized from oleic acid (O) and lauric acid (L), which are the main components of palm oil and palm kernel oil, respectively. The compositions of oleic acid, lauric acid, and their mixtures at molar ratios of 0.5:1 and 1:1 were analyzed using GC-FID. The free fatty acids were classified into saturated, monounsaturated, and polyunsaturated fatty acids, as summarized in Table 2. The feedstock compositions were analyzed in triplicate, and the average values are reported. Lauric acid exhibits a high purity of 99.5%, whereas oleic acid has a purity of 79.4%, containing 10.7% linoleic acid, 4.2% palmitic acid, and 5.8% other minor free fatty acids.

**Table 2:** Composition of Free Fatty Acids in Feedstocks

type of free fatty acid	composition (%)			
	oleic acid (O)	lauric acid (L)	O:L 0.5:1	O:L 1:1
saturated fatty acid	8.0 ± 0.1	100.0 ± 0.0	62.1 ± 1.0	46.3 ± 0.5
capric acid; C10:0	0.0 ± 0.0	0.2 ± 0.0	0.1 ± 0.0	0.1 ± 0.0
lauric acid; C12:0	1.9 ± 0.05	99.5 ± 0.0	59.0 ± 0.9	42.3 ± 0.6
myristic acid; C14:0	0.1 ± 0.0	0.2 ± 0.0	0.2 ± 0.0	0.1 ± 0.1
palmitic acid; C16:0	4.2 ± 0.05		2.0 ± 0.2	2.6 ± 0.1
stearic acid; C18:0	1.8 ± 0.02		0.8 ± 0.03	1.1 ± 0.02
arachidic acid; C20:0	0.1 ± 0.1		0.0 ± 0.02	0.0 ± 0.03
monounsaturated fatty acid	81.1 ± 0.3		33.7 ± 0.7	47.8 ± 0.6
palmitoleic acid; C16:1	0.0 ± 0.05		0.0 ± 0.01	0.0 ± 0.01
oleic acid;*cis*-C18:1	79.4 ± 0.4		32.4 ± 0.9	46.2 ± 1.1
elaidic acid;*trans*-C18:1	1.3 ± 0.1		1.3 ± 0.6	1.4 ± 0.7
eicosenoic acid; C20:1	0.4 ± 0.04		0.1 ± 0.1	0.1 ± 0.1
polyunsaturated fatty acid	10.8 ± 0.3		4.2 ± 0.4	5.9 ± 0.3
linoleic acid; C18:2	10.8 ± 0.3		4.2 ± 0.2	5.9 ± 0.3

### 3.3 Synthesis and Characterization of OL-EST-EH

OL-EST-EH was synthesized under vacuum through two consecutive reactions using Amberlyst-15, a sulfonic acid-functionalized ion-exchange resin, as a heterogeneous catalyst (Scheme 1).

**Scheme 1:**
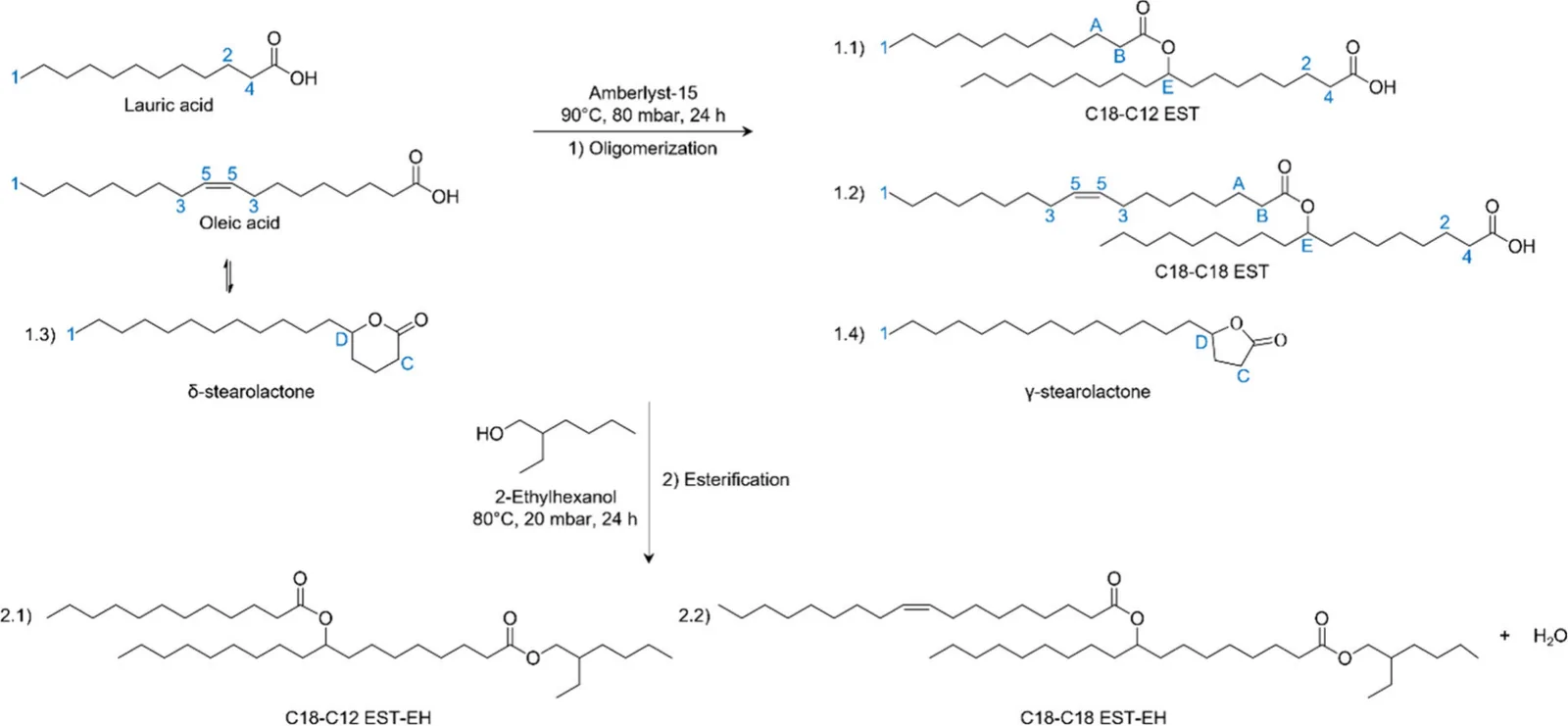
Reaction Pathway of Estolide Ester Synthesis Using Lauric Acid and Oleic Acid as Feedstocks^a^

a Blue labels indicate the positions assigned to the ^1^H NMR chemical shifts.

In the first step, the reaction involved the oligomerization to form estolide linkages, using oleic acid and lauric acid as the base unit and capping acid, respectively. The oligomerization pathway proceeds via nucleophilic addition at the double bond of oleic acid, where protonation by the sulfonic acid group generates a carbocation intermediate. Subsequently, the hydroxyl group of the carboxylic acid from another fatty acid attacks the carbocation, forming estolide linkages, producing C18–C18 EST and C18–C12 EST (1.1 and 1.2 in Scheme 1). This oligomerization is terminated by the incorporation of lauric acid, a saturated fatty acid, as a capping agent to produce C18–C12 EST. Additionally, lactone can be produced as a byproduct via intramolecular cyclization of oleic acid, involving its carboxyl group and the carbocation located at either C-9 or C-10 (1.3 and 1.4 in Scheme 1). The γ-stearolactone (five-membered ring) is generally favored in terms of thermodynamic stability, making it less prone to ring-opening. In contrast, δ-stearolactone (six-membered ring) can revert to oleic acid and shift toward estolide formation.^44^ In the second step, the carboxyl groups of the estolides and the remaining fatty acids were esterified with 2-ethylhexanol (2-EH), resulting in estolide esters (OL-EST-EH) and monoesters (OL-EH). After synthesis, the chemical structures, conversion, and product composition were analyzed using ^1^H NMR, FTIR, and GC-FID.

Proton chemical shifts and their corresponding assignments for both feedstocks and estolide products are summarized in Table 3. The ^1^H NMR spectra provided structural information confirming the formation of the estolide linkage (Scheme 1). The characteristic signal indicating the presence of the estolide linkage appears as a methine proton at 4.92–4.67 ppm. Additionally, the increased signal of the α-methylene proton adjacent to the ester and the decreased α -methylene proton adjacent to the acid were observed at 2.30–2.20 and 2.40–2.30 ppm, respectively. Additional signals include the α-methylene proton shifts at 2.58–2.48 ppm, and the δ-methine proton shifts at 4.52–4.42 ppm adjacent to the carbonyl group in the lactone ring.

**Table 3:** Proton Chemical Shifts (δ, ppm) and Corresponding Functional Groups of Feedstock and OL-EST as Determined by ^1^H NMR Spectroscopy

peak	type of proton	chemical shift (δ, ppm)		attribution
		feedstock	OL-EST	
1	–CH_3_	0.91–0.83	0.92–0.79	proton attached to the terminal carbon
A	–CH_2_CH_2_(C=O)–O–		1.54–1.42	proton attached to the β-carbon of the esters
2	–CH_2_CH_2_(C=O)–OH	1.66–1.56	1.68–1.54	proton attached to the β-carbon of the carboxylic acid
3	–CH_2_–CH=CH-CH_2_–	2.06–1.96	2.06–1.88	allylic proton
B	–CH_2_(C=O)-O–CH_2_–		2.30–2.20	proton attached to the α-carbon of the ester
4	–CH_2_(C=O)–OH	2.37–2.29	2.40–2.30	proton attached to the α-carbon of the carboxylic acid
C	–CH–O–C=O–CH_2_-		2.58–2.48	proton attached to the α-carbon of the stearolactone
D	–O–CH(CH_2_ )-CH_2_CH_2_C(=O)–		4.52–4.42	proton attached to the δ-carbon of the stearolactone
E	–CH–O(C=O)–CH_2_–		4.92–4.76	EST linkage
5	–CH=CH–	5.41–5.28	5.49–5.25	unsaturation protons

FTIR was employed to characterize the functional groups of feedstock and products (Figure 1). The peak at 3008 cm^–1^ corresponds to C–H stretching of an unsaturated free fatty acid. The other peaks at 2917–2849 cm^–1^ correspond to the C–H stretching of an aliphatic hydrocarbon chain, while peaks located at 1704 and 938 cm^–1^ are assigned to the C=O stretching band and O–H bending of carboxylic acid, respectively. The vibration band at 722 cm^–1^ was attributed to the overlapping of −CH_2_ rocking and *cis*-C=C– out-of-plane of olefins.^45^ After oligomerization for 24 h, the FTIR spectra showed a decrease in the =C–H stretching band at 3008 cm^–1^ of unsaturation, indicating the reduction of unsaturation. Meanwhile, the C=O stretching band of the carboxylic acid at 1704 cm^–1^ shifted to a new C=O stretching of the ester group corresponding to the estolide linkages. Additionally, a vibration at 966 cm^–1^ was observed in the products obtained from the high-temperature reaction, corresponding to *trans*-C=C bending, which suggests *cis*–*trans* isomerization of the remaining unsaturated fatty acids. The resulting *trans* isomers possess a less reactive structure, resulting in a slower rate of estolide formation and potentially yielding different products. These unsaturated fatty acids were more likely to form stearolactone byproducts, as indicated by the peak observed at approximately 1782 cm^–1^, which is attributed to the carbonyl stretching (C=O) of the lactone ring.^46,47^

**Figure 1:**
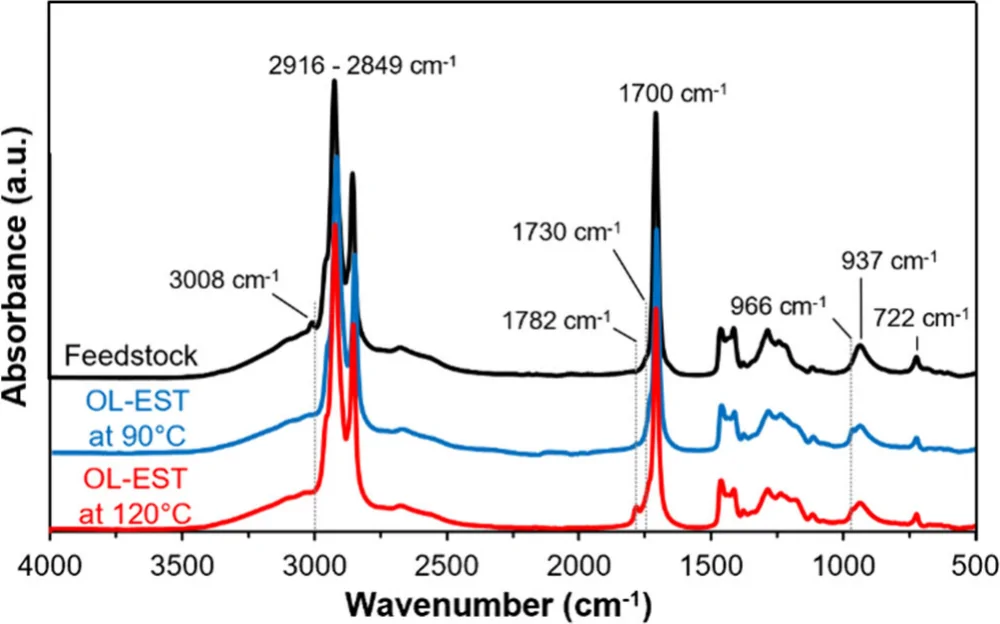
FTIR spectra of a mixture of oleic acid and lauric acid feedstock and OL-EST. Reaction conditions: O:L = 1:1, 20 wt % Amberlyst-15 under a vacuum of 80 mbar at 90 °C (OL-EST at 90 °C) and 120 °C (OL-EST at 120 °C).

GC-FID was used to identify the products and quantify their composition. The overlaid chromatograms of feedstock and representative products are shown in Figure 2. In the feedstock, major peaks were observed at retention times of approximately 15.5 and 25.5 min, corresponding to lauric acid and oleic acid, as confirmed by GC-MS. After oligomerization, a significant reduction in the oleic acid peak was observed, along with the appearance of additional peaks at longer retention times, approximately 34.5 and 37.4 min, which were assigned to C18–C12 EST and C18–C18 EST, respectively. During the subsequent esterification, the carboxyl groups of estolides and fatty acids were esterified with 2-EH to form estolide esters and fatty acid monoesters, collectively referred to as crude OL-EST-EH. The absence of peaks corresponding to estolides and free fatty acids indicated that esterification was complete. Further purification by vacuum distillation to remove monoesters yielded dis.OL-EST-EH, in which the absence of C12-EH and a dramatic decrease in C18-EH confirmed the production of high-purity estolide esters.

**Figure 2:**
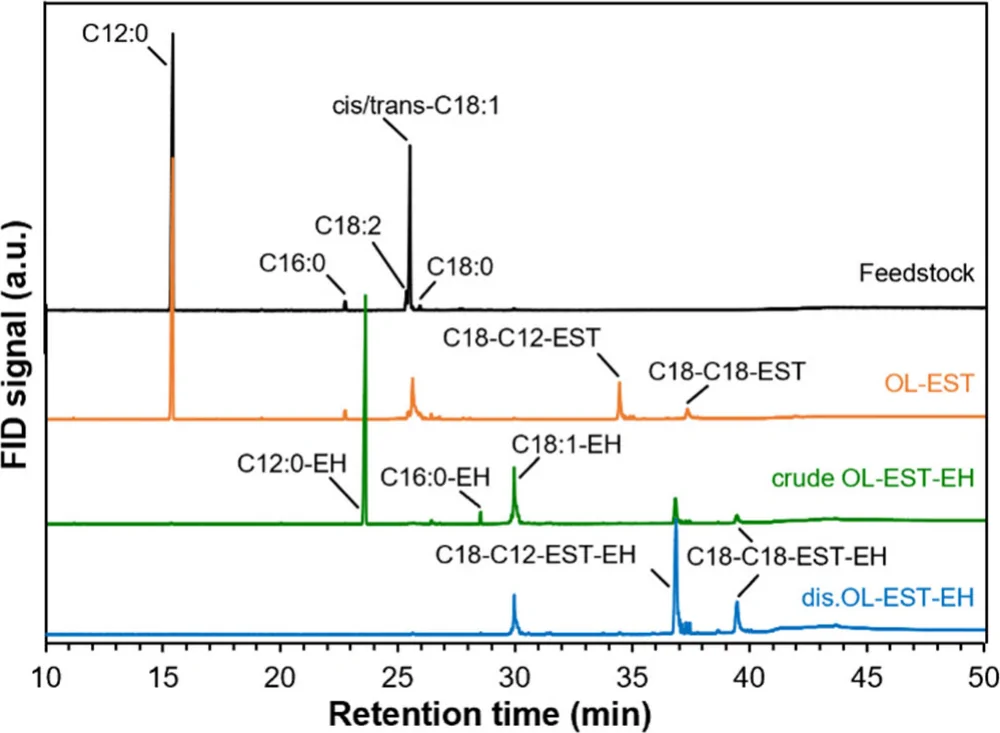
GC-FID chromatography of feedstock and representative products.

For estolide composition analysis, both ^1^H NMR and GC-FID techniques were employed. From ^1^H NMR spectra, the integration of the α-methylene proton signals corresponding to both the acid and ester functionalities was used to calculate α-ester/α-acid ratio, which determined the EN. The EN number was then used to calculate % estolide and compared with the GC-FID result (Table 4). The finding indicated that both analytical techniques provided comparable estolide yields, confirming the reliability of the measurements.

**Table 4:** Calculated Estolide Composition (%) from GC-FID and ^1^H NMR Analysis

O:L	time (h)	GC-FID	^1^H NMR	
		% estolide	EN	% estolide (calculated from EN)
0.5:1	3	6.1	0.043	7.5
	6	11.3	0.059	11.2
	24	26.9	0.128	22.5
1:1	3	15.3	0.075	14.6
	6	21.6	0.104	18.1
	24	35.0	0.179	31.6
	48	37.2	0.221	35.7

#### 3.3.1 Effect of Temperature

The OL-EST was synthesized at various reaction temperatures, and the conversion and product composition were analyzed by GC-FID. The results were categorized into unreacted fatty acids and products, including estolides and lactone byproducts, as shown in Figure 3. The reactions were conducted for 24–48 h at temperatures ranging from 60 to 120 °C. The results indicated that the reaction rate increased rapidly during the first 6 h of the reaction. The conversion of saturated and unsaturated fatty acids increased with increasing reaction temperature (Figure 3a). At 90 °C, the total estolide reached 34.0% after 24 h, but then plateaued due to increased lactone byproduct formation, as shown in Figure 3b. Analysis of *cis*/*trans* composition of C18:1 revealed that *cis*-C18:1, the natural configuration in feedstock, was converted into both estolide and *trans*-C18:1, with a minor amount of *cis*-C18:1 remaining at 24 h (Figure S2). The presence of *trans*-C18:1 at this stage suggests that it is more prone to form lactone via intramolecular cyclization rather than undergoing further intermolecular estolide formation. Consequently, extending the reaction time beyond 24 h did not result in additional estolide formation. At 120 °C, estolide formation reached a maximum at 6 h before declining, while lactone formation increased significantly, attributed to ring closure from unreacted *trans*-C18:1 and the reverse reaction of estolide. Lactone formation reached a high yield of 24.5% at 24 h. Therefore, the reaction at 90 °C was selected as the optimal temperature to minimize lactone formation.

**Figure 3:**
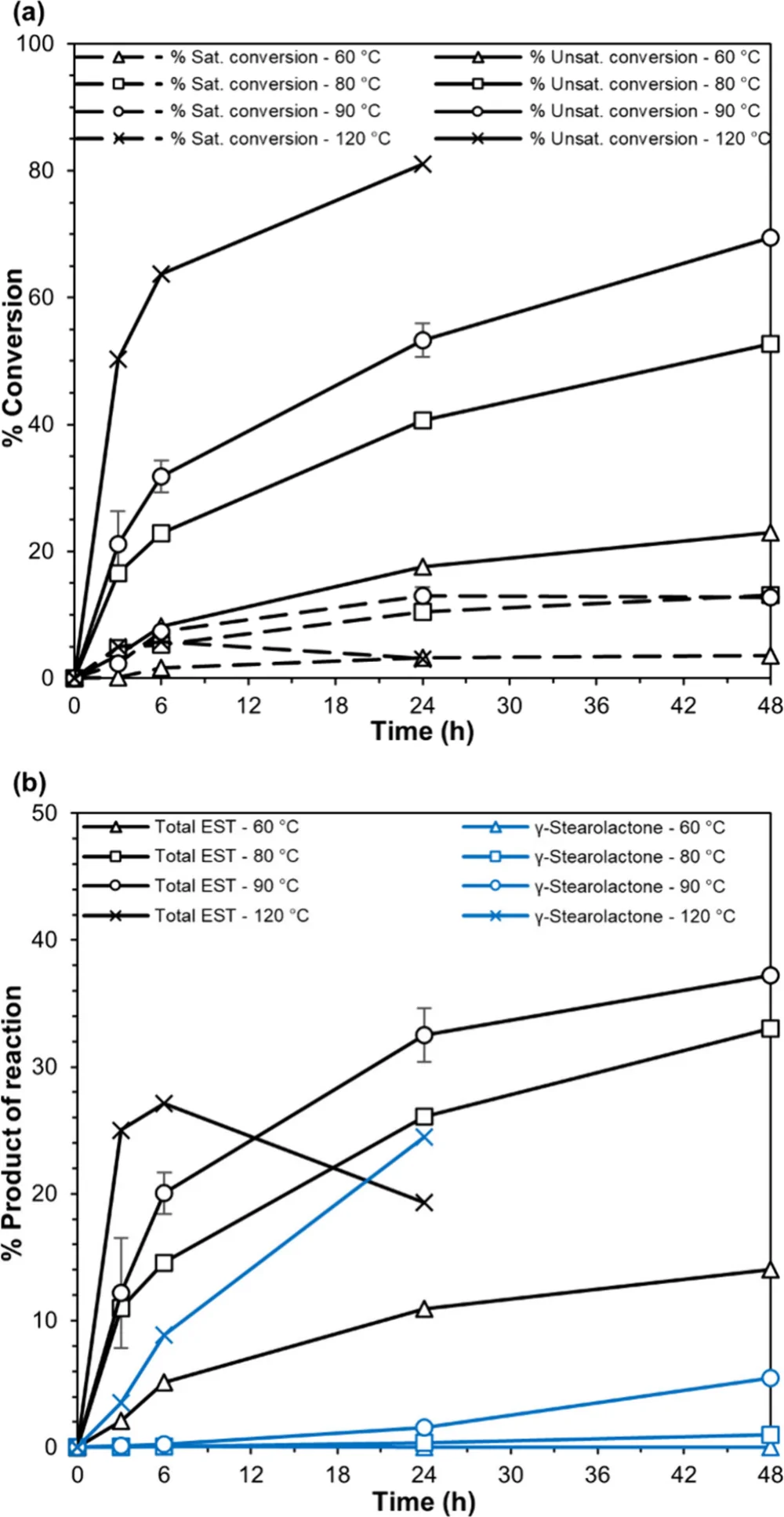
Effect of temperature on the oligomerization process: (a) % conversion of saturated and unsaturated fatty acid; (b) % total EST and γ-stearolactone. Reaction conditions: O:L = 1:1 used 20 wt % Amberlyst-15 under a vacuum of 80 mbar at 60, 80, 90, and 120 °C.

To gain further insight into the reaction mechanism, the experimental GC-FID conversion data obtained during estolide formation were analyzed using kinetic modeling. Based on a previous kinetic study, in which oleic estolide formation was successfully described by an integrated second-order kinetic model, the present experimental data were fitted using the same kinetic model.^48^ The reaction rate constant (*k*) was determined by fitting the experimental data to the apparent second-order rate equation:

$$\frac{-d\left[\mathrm{FFA}\right]}{dt}={k\left[\mathrm{FFA}\right]}^{2}$$

The integrated equation is given by

$$\frac{1}{{\left[\mathrm{FFA}\right]}_{t}}=kt+\frac{1}{{\left[\mathrm{FFA}\right]}_{0}}$$

where [FFA]_0_ and [FFA]_t_ are the initial and time-dependent concentrations of free fatty acids determined by GC-FID, respectively, and *k* is the second-order rate constant.

The linear graphs of 1/[FFA]_t_ versus reaction time at various temperatures are presented in Figure S3a, where the slope represents the rate constant. The corresponding second-order rate constants (*k*) and coefficients of determination (*R* ^2^) obtained from linear regression analysis are summarized in Table S3. The second-order rate constant increased from 4.9 × 10^–5^ to 1.8 × 10^–4^ normalized peak/area·h with increasing reaction temperature, indicating that elevated temperatures accelerated the estolide formation reaction. At 90 °C, the calculated rate constant was 1.8 × 10^–4^, which is comparable to the previously reported value of 2.56 × 10^–4^ for the conventional synthesis of oleic estolide reaction. The slight difference can be reasonably attributed to the use of a different catalytic system and reaction conditions. Nevertheless, both values are of the same order of magnitude, suggesting comparable kinetic behavior. To further evaluate the effect of temperature on the reaction rate, the Arrhenius equation was applied:

$$\ln k=-\frac{E_{a}}{RT}+\ln A$$

where *E*_a_ is the activation energy (J/mol), *R* is the universal gas constant (8.314 J/mol·K), and *T* is the absolute temperature (K).

The Arrhenius plot of ln *k* versus 1/*T* is shown in Figure S3b, exhibiting a good linear relationship over the investigated temperature range. The activation energy (*E*_a_) was determined from the slope of the fitted line (−*E*_a_/*R*) and calculated to be 42.93 kJ/mol. The calculated activation energy (42.93 kJ/mol) suggests that Amberlyst-15 effectively facilitates estolide formation under the investigated reaction conditions.

#### 3.3.2 Effect of the Mole Ratio

The product structure is significantly influenced by the molar ratio of the feedstock, which, in turn, affects its physicochemical properties. Various molar ratios of oleic acid to lauric acid (0.25:1 to 1:0) were employed for estolide synthesis using 10 wt % of Amberlyst-15 at 90 °C and 80 mbar. The use of excess lauric acid (0.25:1) resulted in a higher product selectivity toward C18–C12 EST of 77% (Figure 4). However, the total percentage of EST was lowered compared to the other mole ratios due to the limited amount of oleic acid available to serve as the base unit for chain extension with lauric acid (capping unit). Increasing the oleic acid ratio resulted in higher selectivity for C18–C18 EST, although the overall estolide content remained approximately 22–23%. These findings indicate that the specificity of the estolide structure is strongly dependent on the molar ratio of the reactants. The result suggests that the molar ratio can serve as a key parameter for controlling estolide structure. For the synthesis of estolides with an estimated EN = 1, a molar ratio of 0.5:1 or lower is recommended. In case of estolide EN ≥ 2, oleic acid should be used as a starting material, with saturated fatty acid added later as a capping agent when the desired estolide chain length has been achieved.

**Figure 4:**
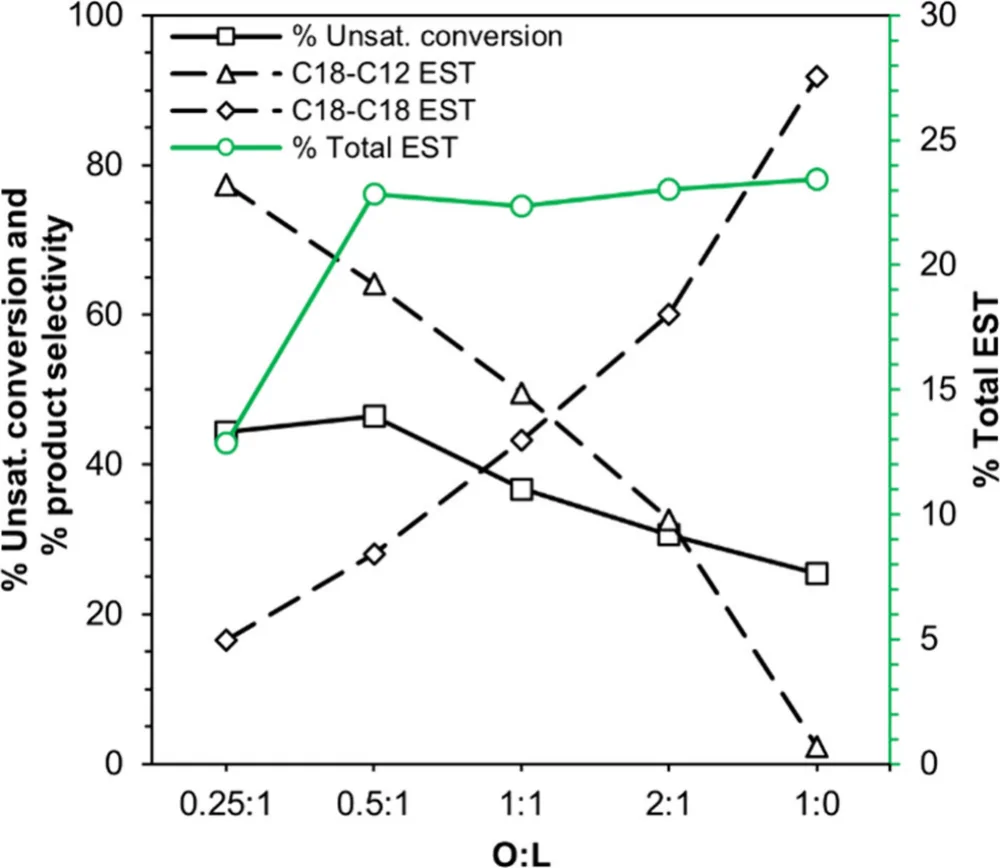
Effect of lauric/oleic acid mole ratio on unsaturated fatty acid conversion and product distribution, including total estolide, C18–C12 EST, and C18–C18 EST. Reaction conditions: 10 wt % Amberlyst-15 at 90 °C under a vacuum of 80 mbar for 24 h.

#### 3.3.3 Effect of the Catalyst Amount

This study investigated the effect of catalyst amount on estolide synthesis using a 1:1 molar ratio of oleic acid to lauric acid at a temperature of 90 °C. The GC-FID results indicate that the conversion rates of saturated and unsaturated fatty acids increased over time, correlating with the quantities of C18–C12 EST and C18–C18 EST produced (Figure 5). In the initial 6 h, the reaction rates of C18–C12 EST and C18–C18 EST were similar, with an increase in rate corresponding to a higher catalyst quantity. Subsequently, the C18–C18 EST was slightly increased due to the increase in γ-stearolactone, leading to enhanced conversion of unsaturated fatty acids. The result showed that as the catalyst amount increased, the product yield also increased, with total EST at 24 h being 21.8%, 34.0%, and 39.4%, respectively. These results showed that increasing the catalyst amount from 20% to 30% did not significantly enhance the total EST due to unsaturated fatty acid conversion leading to byproducts. Therefore, the amount of catalyst at 20 wt % was sufficient for the synthesis of estolide from oleic acid and lauric acid, because the use of a lower or higher catalyst will affect the estolide and lactone formed. Therefore, a catalyst loading of 20 wt % was selected for the synthesis of estolide ester to analyze product properties at oleic-to-lauric molar ratios of 0.5:1 and 1:1.

**Figure 5:**
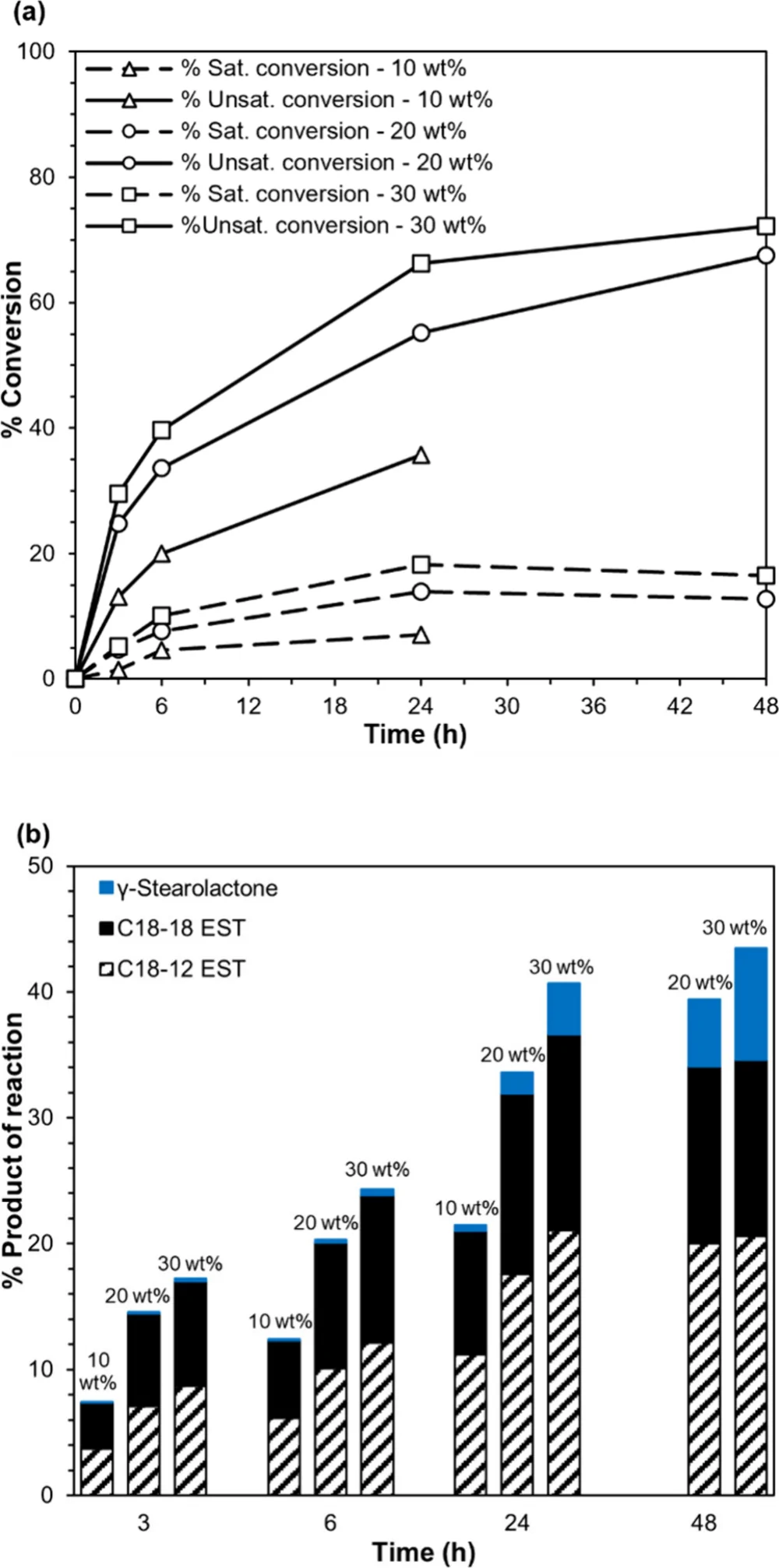
Effect of the catalyst amount on the oligomerization reaction: (a) % conversion of saturated and unsaturated fatty acid; (b) % product of reaction consisting of C18–C12 EST, C18–C18 EST, and γ-stearolactone. Reaction conditions: O:L = 1:1, temperature 90 °C under a vacuum of 80 mbar with Amberlyst-15 10–30 wt %.

### 3.4 Biolubricant Properties

The biolubricant properties of the synthesized estolide esters were characterized and are summarized in Table 5. The crude OL-EST-EH consisted of estolide esters and the monoalkyl esters (light fraction). To remove the light fraction, further distillation was performed, yielding distilled OL-EST-EH (dis.OL-EST-EH) and monoalkyl esters (OL-EH). The TAN of the products ranged from 0.5 to 2.1 mg KOH/g, indicating successful conversion of carboxyl groups to ester groups via esterification. All the synthesized ester products exhibited excellent viscosity index (VI) values greater than 150, consistent with the characteristic properties of ester-based lubricants. The mole ratio of fatty acids influenced both the structures and physicochemical properties of the products. Specifically, OL-EST-EH synthesized at a 0.5:1 oleic-to-lauric ratio showed lower viscosity than the 1:1 ratio, attributed to its higher proportion of C12–C18 EST-EH, which contains a shorter capping chain compared to C18–C18 EST-EH (Table 6). After the distillation to remove the light fraction, the purified estolide esters (dis.OL-EST-EH) yielded 85.0–87.8% purity and exhibited higher viscosity than the crude product. dis.OL-EST-EH prepared at mole ratios of 0.5:1 and 1:1 showed viscosities of 36.50 and 43.64 cSt, corresponding to an ISO VG 32 and 46, respectively. The dis.OL-EST-EH (0.5) exhibited a lower PP (below −30 °C), suggesting that C12–C18 EST-EH has a lower melting point than C18–C18 EST-EH. Differential scanning calorimetry (DSC) analysis was performed to investigate the crystallization and melting behaviors of OL-EST-EH (0.5) and related products, in comparison with palm olein (POL), as shown in Figure 6. The corresponding thermal parameters, including peak temperature (*T*_p_), onset temperature (*T*_o_), and endset temperature (*T*_e_), are summarized in Table 7. The crystallization temperatures of OL-EST-EH (0.5) and its related products exhibited similar thermal transitions, consistent with the PP results. The melting profile of OL-EH displayed two distinct peaks (*T*_p_ = −37.84 and −44.69 °C), corresponding to the melting of oleic ethylhexyl ester and lauric ethylhexyl ester, respectively. In contrast, its crystallization profile showed only a single peak (*T*_p_ = −49.89 °C), suggesting that lauric ethylhexyl ester inhibited the crystallization of oleic ethylhexyl ester, thereby shifting the crystallization to a lower temperature. The low-temperature behavior of OL-EH was mostly similar to that of OL-EST-EH, indicating its potential application as a biosolvent for cosmetic formulations, detergents, and cleaning products, as well as a blending component in biobased lubricants to provide a range of viscosities suitable for different applications. Palm olein, although derived from the unsaturated fraction of palm oil, is predominantly composed of palmitic and oleic acids and exhibits significantly higher crystallization and melting temperatures. Moreover, its DSC thermogram displayed multiple broad thermal transitions, reflecting the complex triglyceride structure with the presence of various fatty acids.^28^ Based on the DSC analysis, it can be suggested that the synthetic esters exhibited more favorable thermal properties for low-temperature applications in industrial lubrication, whereas palm oil shows certain limitations in low-temperature performance.

**Table 5:** Physicochemical Properties of OL-EST-EH, OL-EH, Palm Olein, Soybean Oil, and Polyol Esters

sample	oleic:lauric	properties							
		viscosity (mm^2^/s)		VI	PP (°C)	CP (°C)	OS (h) at 140 °C	TAN (mg KOH/g)	ref
		at 40 °C	at 100 °C						
crude OL-EST-EH (0.5)	0.5:1	8.53	2.64	158	–32	–30	7.1	0.678	this work
crude OL-EST-EH (1)	1:1	11.09	3.18	163	–27	–25	6.9	0.935	this work
dis.OL-EST-EH (0.5)	0.5:1	36.50	7.28	169	–33	–30	20.3	1.286	this work
dis.OL-EST-EH (1)	1:1	43.64	8.30	169	–30	–29	7.2	2.123	this work
OL-EH ester (0.5)	0.5:1	5.99	2.04	153	–32	–30	1.5	0.579	this work
OL-EH ester (1)	1:1	6.69	2.21	154	–25	–23	0.9	1.289	this work
Natural and Synthetic Esters									
palm olein (POL)		40.26	8.39	191	4	8	3.7	0.312	this work
soybean oil (SO)		32.0	7.65	221	–8	–4	1.2	0.109	this work
NPG-PFAD		25.55	5.57	166	18		3.0		^9^
TMP-PFAD		42.5	8.61	187	12		17.5		^9^
TMP triester (refined and used soybean oils)		34.4–36.6			–11 to −9			1.8–1.5	^51^
Castor NPG esters		116.8–133.1	11.3–12.6	79–84	–24 to −27				^52^
Commercial Synthetic Esters									
PANOLIN S4 HLP Synth EAL32 (Shell)		33.30	6.50	164	–54				^53^
Dehylub Series (Emry Oleochemicals)		14–150		80–200	–45 to +10			≤2	^54^
Mobil SHC 525		46	8.54	154	–54				^55^

**Table 6:** Product Composition and Selectivity of dis.OL-EST-EH

product	dis.OL-EST-EH (0.5)	dis.OL-EST-EH (1)
estolide ester composition (wt %)	85.0 ± 1.1	87.8
product selectivity (%)		
lactones	0.02 ± 0.3	0.07
estolide	1.0 ± 0.5	0.9
EH ester	17.0 ± 0.3	13.0
- C12-EH ester	0.0	0.0
- C18-EH ester	16.0 ± 0.2	12.4
mono estolide ester (EN = 1)	81.9 ± 0.8	86.0
- C18–C12 EST-EH	59.4 ± 0.6	48.4
- C18–C14 EST-EH	0.4 ± 0.01	0.3
- C18–C16 EST-EH	1.1 ± 0.01	1.8
- C18–C18 EST-EH	19.7 ± 0.2	33.7
dimer estolide ester (EN = 2)	1.5 ± 0.4	1.8

**Figure 6:**
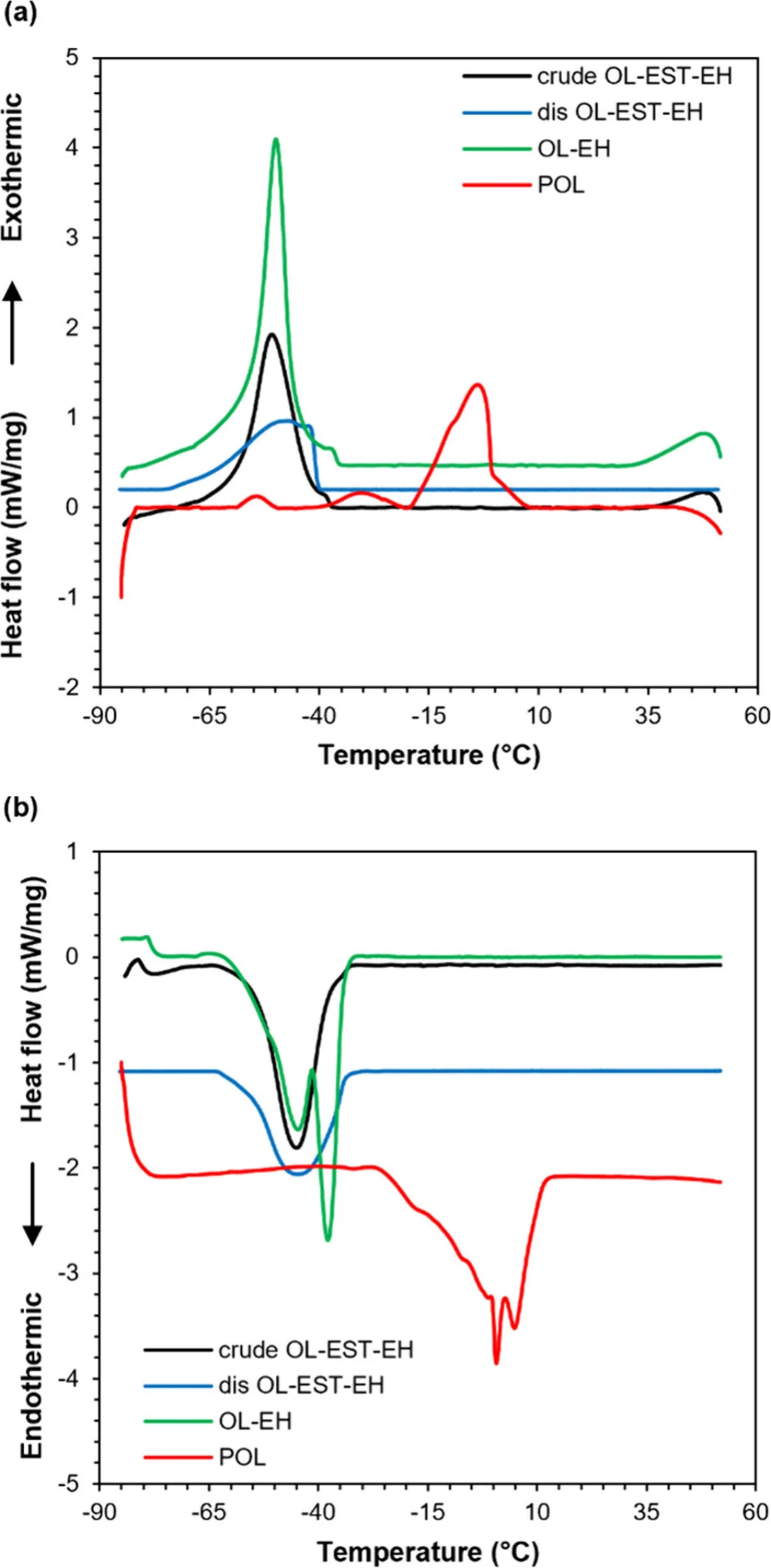
DSC spectra of base oils from (a) cooling and (b) heating scans.

**Table 7:** DSC Data of Base Oils

	cooling scan (crystallization)		
sample	*T*_peak_ (°C)	*T*_onset_ (°C)	*T*_endset_ (°C)
crude OL-EST-EH	–50.75	–41.71	–58.03
dis.OL-EST-EH	–47.20	–40.27	–67.25
OL-EH	–49.89	–45.74	–54.70
POL	–54.33, −30.57, −3.92	7.31	–58.71

	heating scan (melting)		
sample	*T*_peak_ (°C)	*T*_onset_ (°C)	*T*_endset_ (°C)
crude OL-EST-EH	–45.13	–53.7	–37.52
dis.OL-EST-EH	–44.95	–56.39	–33.87
OL-EH	–44.69, −37.84	–53.26	–34.37
POL	0.57, 4.73	–20.2	10.06

Incorporation of saturated-capped estolides improved oxidative stability because the synthesized estolide esters contain fewer double bonds that are susceptible to oxidation. As expected, the oxidation stability of dis.OL-EST-EH (0.5) was superior to that of dis.OL-EST-EH (1), which can be attributed to its higher proportion of saturated-capped estolide ester, specifically C18–C12 EST-EH (59.4%), and its lower content of unsaturated-capped C18–C18 EST-EH (19.7%) (Table 6). Soybean oil exhibited a lower pour point than POL (Table 5), indicating better low-temperature performance and suitability as a base oil in lubricant formulations. However, its practical application is limited by its low oxidative stability (OS = 1.2 h), which restricts its use under mild thermo-oxidative exposure.^49,50^ In contrast, POL showed higher oxidative stability (3.7 h) but remained unsuitable for lubricant applications due to its relatively high pour point. Furthermore, upon thermolysis, triglycerides tend to degrade into di- and monoglycerides, which exhibit even higher pour points. The synthesized dis.OL-EST-EH (0.5) offers a promising solution, significantly improving low-temperature fluidity (−30 °C) and oxidative stability (20.3 h), demonstrating its potential as a high-performance biolubricant base oil. A comparison with representative biolubricant base oils reported in the literature (Table 5) further indicates that the lubricant properties of OL-EST-EH are comparable to those of chemically synthesized polyol esters. In addition, a comparison with commercially available synthetic esters demonstrates that the synthesized estolide ester exhibits a competitive viscosity index, favorable low-temperature fluidity, and good oxidative stability, highlighting its potential as a sustainable lubricant base oil. Moreover, compared with polyol esters derived from palm fatty acid distillate (PFAD), dis.OL-EST-EH (0.5) demonstrates superior performance in terms of both oxidative stability and pour point. Although free fatty acid was used as a model feedstock in this study, the proposed two-step process demonstrates an effective approach for producing high-value biolubricant base oils from renewable fatty acids through estolide formation followed by esterification. Excess 2-ethylhexanol is separated during purification and could potentially be recovered for reuse, while the monoester fraction obtained from vacuum distillation may find future application as biosolvents. The reusability of the heterogeneous Amberlyst-15 catalyst further supports the sustainability of the process. Overall, these findings provide a basis for extending the process to renewable and waste-derived palm oil feedstocks in future studies, contributing to more efficient utilization of biomass resources for sustainable biolubricant production.

## 4 Conclusion

This study successfully synthesized estolide esters from oleic acid and lauric acid, the primary fatty acids derived from palm oil and palm kernel oil, using Amberlyst-15 as a solid acid catalyst. The integrated one-pot sequence, comprising estolide formation followed by esterification, enabled efficient upgrading of palm-derived fatty acids while maintaining catalyst stability. After two consecutive reaction steps, only a slight decrease in ion-exchange capacity was observed, and the overall chemical composition of the catalyst remained unchanged. Compared with conventional mineral acid catalysts, Amberlyst-15 offers practical advantages, including facile separation, reduced corrosiveness, and a lower-waste process.

Under optimized conditions, estolide formation achieved a yield of 34%, and subsequent esterification with 2-ethylhexanol proceeded to > 95% conversion to a mixture of estolide esters and fatty acid esters (TAN < 2 mg KOH/g). The incorporation of lauric acid as a capping unit significantly enhanced the physicochemical properties of the products, particularly low-temperature performance, resulting in PP of −32 °C for crude OL-EST-EH (0.5) and −33 °C for dis.OL-EST-EH (0.5). In addition, the resulting esters also exhibited high oxidative stability due to their low degree of unsaturation and maintained high viscosity indices (>150) with kinematic viscosities ranging from 8 to 43 mm^2^/s. This wide viscosity range demonstrates their versatility and suitability for a range of lubricant applications.

The synthesized estolide esters display favorable lubricating characteristics, including excellent low-temperature fluidity, high oxidative stability, and a broad viscosity range. These findings highlight the potential of this heterogeneous catalytic approach as a sustainable strategy for producing biobased lubricant base oils from palm-derived fatty acids.

## Supplementary Material



## Notes

SEM images of Amberlyst-15 for fresh catalyst, catalyst after oligomerization (first step), and catalyst after esterification (second step), recorded at magnifications of 35×, 120×, and 12,000×, respectively (Figure S1), elemental compositions of the fresh and used Amberlyst-15 catalysts determined by SEM-EDS (Table S1), product composition after reuse of Amberlyst-15 in estolide ester reaction (Table S2), *cis*–*trans* isomerization of C18:1 as a function of the reaction times, with reaction conditions of O:L = 1:1 under a vacuum of 80 mbar, 90 °C, and 20 wt % Amberlyst-15 (Figure S2), kinetic study of estolide formation with plot of 1/[FFA]_t_ versus time at different temperature reactions and (b) Arrhenius plot for determination of the activation energy (Figure S3), and rate constants at different temperature reactions (Table S3) (PDF)
