# Supplementary material for: Catalytic Valorization of Palm-Based Fatty Acids into High-Performance Estolide Ester Lubricants
Source: ACS Omega. 2026 Sep 24;11(39):59378–90. doi: 10.1021/acsomega.6c05480 (PMC13712477; doi:10.1021/acsomega.6c05480)
Supplement: Supplementary file 1 [file ao6c05480_si_001.pdf]

Supporting Information

Catalytic Valorization of Palm-Based Fatty Acids into High-Performance Estolide Ester Lubricants

Paphada Watcharapo,[a] Pawnprapa Pitakjakpipop,[a] Wipark Anutrasakda,[b] and Harit Pitakjakpipop[c]*

[a]National Energy Technology Center (ENTEC), National Science and Technology Development Agency (NSTDA), 114 Thailand Science Park, Phahonyothin Road, Klong 1, Klong Luang, Pathum Thani 12120, Thailand

[b]Department of Chemistry, Faculty of Science, Chulalongkorn University, Payathai Road, Patumwan, Bangkok 10330, Thailand

[c]National Nanotechnology Center (NANOTEC), National Science and Technology Development Agency (NSTDA), 111 Thailand Science Park, Phahonyothin Road, Klong 1, Klong Luang, Pathum Thani 12120, Thailand

*Corresponding author.

E-mail: Pawnprapa.pit@entec.or.th

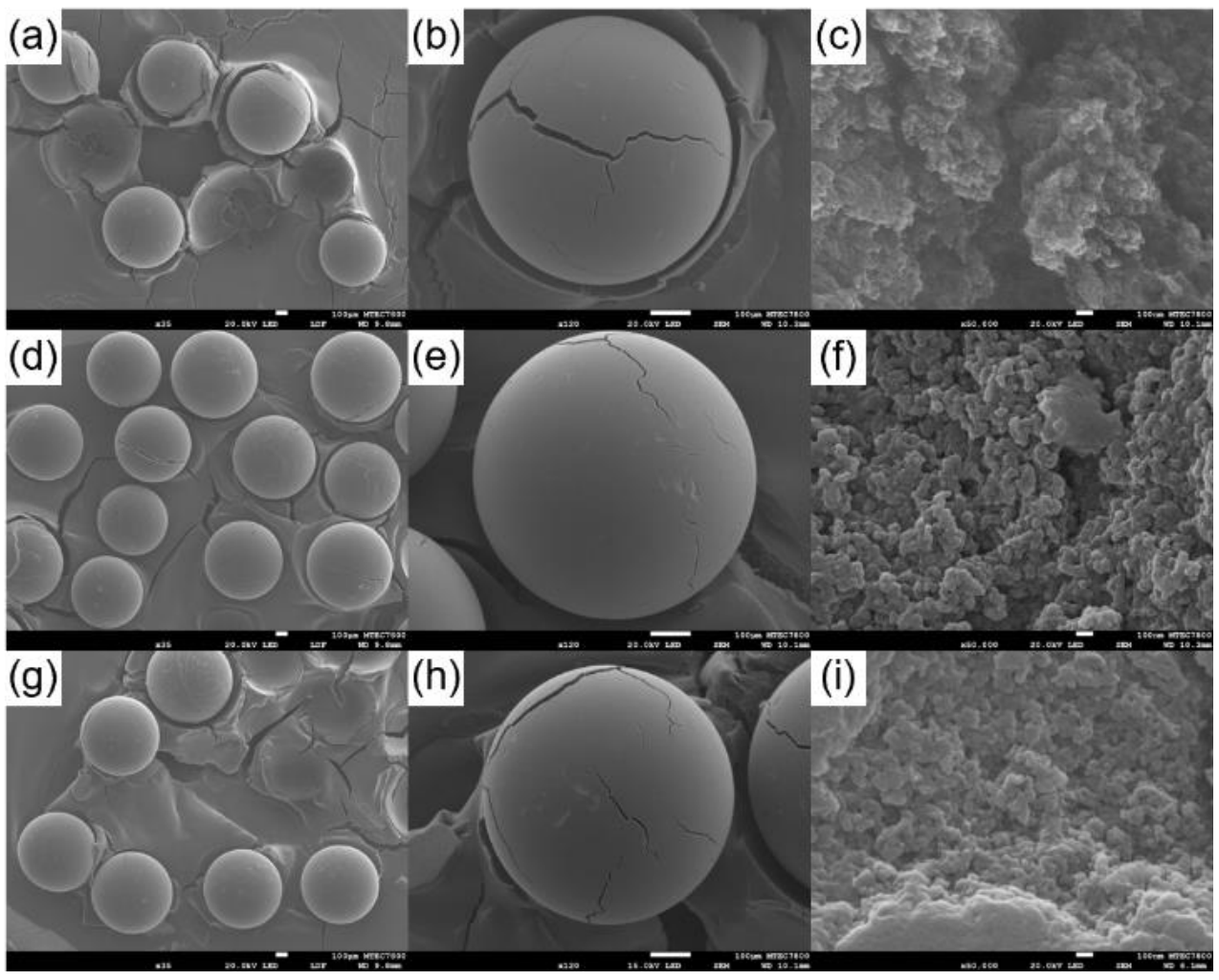

Figure S1. SEM images of Amberlyst-15: (a-c) fresh catalyst, (d-f) catalyst after oligomerization (1st step), and (g-i) catalyst after esterification (2nd step), recorded at magnifications of 35x, 120x, and 12,000x, respectively.

Table S1 Elemental compositions of the fresh and used Amberlyst-15 catalysts determined by SEM-EDS.

Element (wt%)	Fresh	Used 1st step	Used 2nd step
C	56.56 ± 0.63	57.75 ± 0.07	57.74 ± 0.31
O	27.05 ± 0.18	26.81 ± 0.69	26.63 ± 0.43
S	16.40 ± 0.45	15.45 ± 0.76	15.64 ± 0.74
Total	100.00	100.00	100.00

Table S2 Product composition after reuse of Amberlyst-15 in estolide ester reaction.

%Composition	1st cycle	2nd cycle	3rd cycle
Free fatty acids	1.30	1.11	1.44
Lactones	1.20	3.75	4.60
Estolides	0.11	0.37	0.78
EH esters	70.15	66.99	64.19
Estolide esters	27.24	27.78	28.99

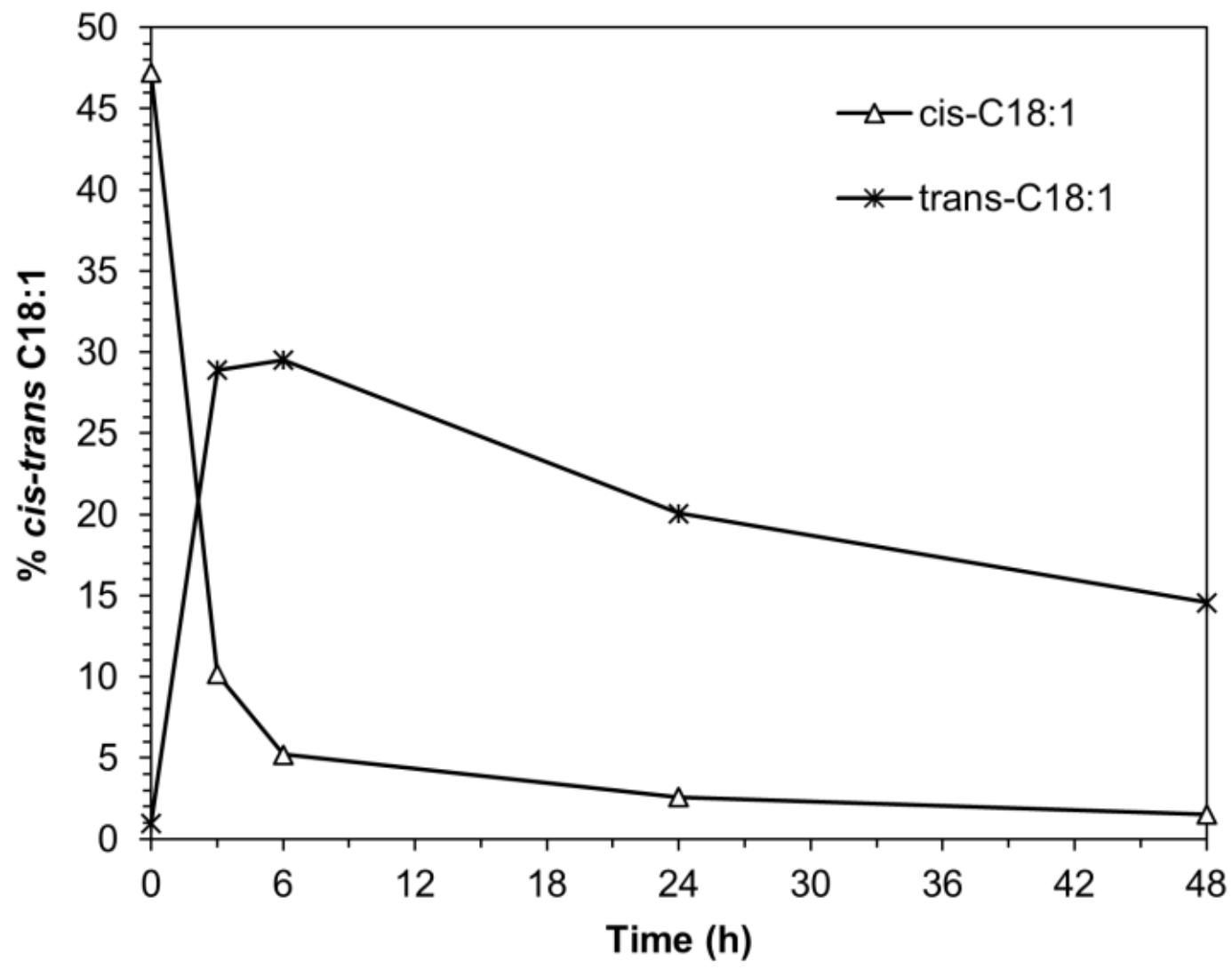

Figure S2. *Cis-trans* isomerization of C18:1 as a function of reaction times, Reaction conditions: O:L = 1:1 under a vacuum of 80 mbar, 90 °C, 20 wt% Amberlyst-15.

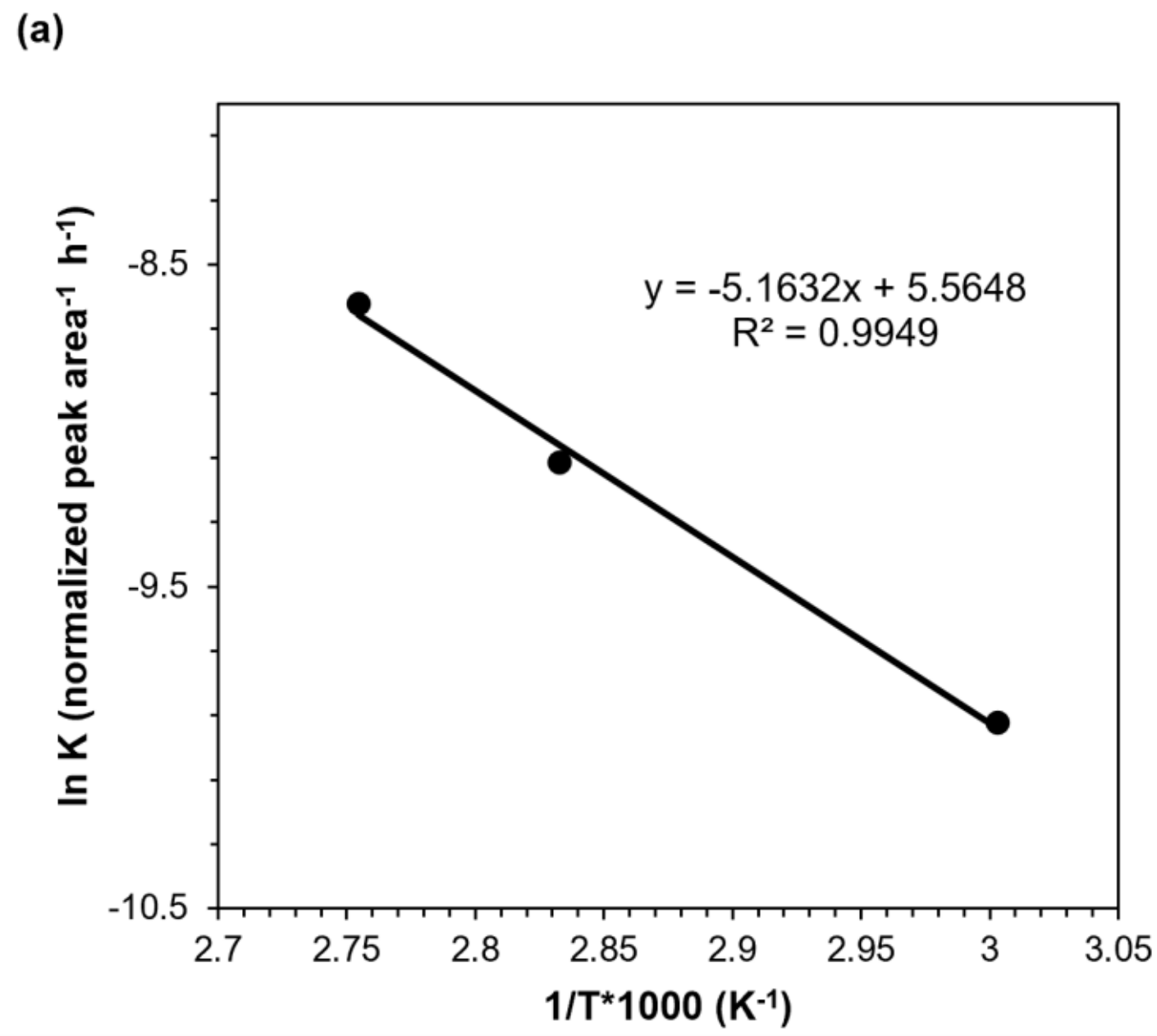

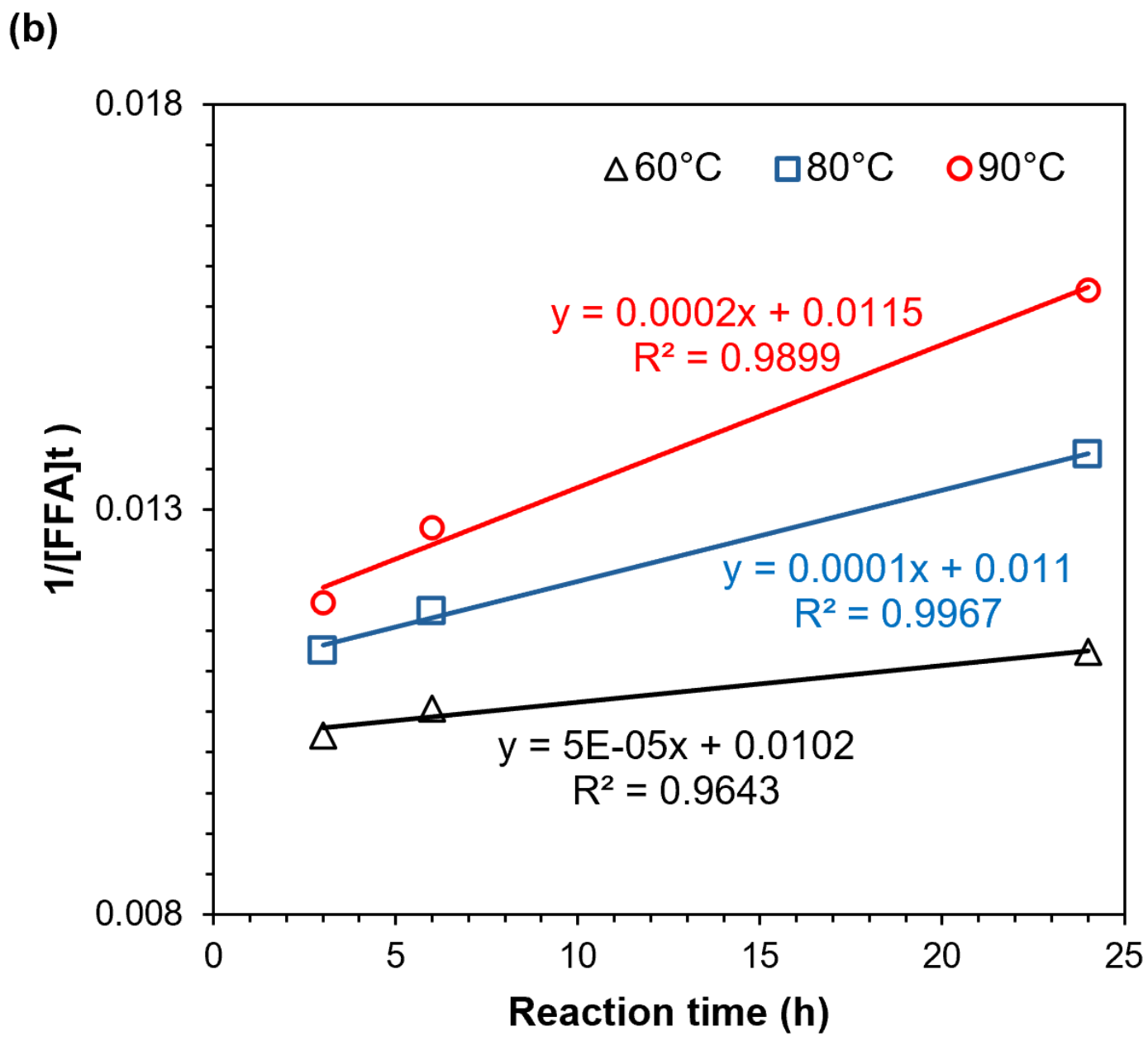

Figure S3. Kinetic study of estolide formation. (a) plot of $1/[FFA]_t$ versus time at different temperature reaction. (b) Arrhenius plot for determination of activation energy.

Table S3 Rate constants at different temperatures reaction.

Temperature	Rate constant (k) (normalized peak area^{-1} h^{-1})	R^2
60 °C	4.9×10^{-5}	0.9643
80 °C	1.1×10^{-4}	0.9967
90 °C	1.8×10^{-4}	0.9899
